# Assessing the Impact of a Reality TV Fashion Model Contest on Women's State Body Dissatisfaction, Affect, and Self‐Esteem: An Experience Sampling Study of Women With and Without Eating Disorders

**DOI:** 10.1002/erv.3185

**Published:** 2025-03-05

**Authors:** Friederike‐Johanna Holtmann, Christopher Lalk, Julian Rubel, Silja Vocks

**Affiliations:** ^1^ Department of Clinical Psychology and Psychotherapy Institute of Psychology Osnabrück University Osnabrück Germany; ^2^ Department of Clinical Psychology and Psychotherapy of Adulthood Institute of Psychology Osnabrück University Osnabrück Germany

**Keywords:** body dissatisfaction, body image, eating disorders, negative affect, thin‐ideal media

## Abstract

**Objective:**

Reality TV model contests like Germany's Next Topmodel (GNTM) remain popular worldwide, but face media criticism for potentially impacting women's body image. This experience sampling study is the first to examine GNTM's impact on body dissatisfaction, affect, and self‐esteem among women with and without eating disorders (EDs).

**Method:**

Women with (*n* = 36) and without self‐reported EDs (*n* = 143) watched the 18^th^ season of GNTM in their private environment to enhance ecological validity. Questionnaires on body dissatisfaction, body‐related self‐ideal discrepancy, affect, and self‐esteem were completed before, during, and after each episode. Statistical analysis was conducted using Bayesian multilevel modelling.

**Results:**

After watching an episode, women with self‐reported EDs showed a significantly stronger increase in body dissatisfaction, self‐ideal discrepancy, and negative affect compared to women without EDs (*p* < 0.001). Moreover, in women with EDs, the self‐ideal discrepancy became significantly greater over the course of the entire season (*p* < 0.01).

**Conclusions:**

This study demonstrates that TV model contests may have negative psychological effects on women, particularly women with EDs. Accordingly, these shows may play a role in the aetiology and maintenance of EDs. Interventions increasing the critical distance to such content might be developed for at‐risk groups.

**Summary**

- This study provides first evidence that women with and without eating disorders show increased body dissatisfaction after watching an episode of the TV model contest Germany's Next Topmodel.
- Additionally, women with eating disorders showed increased negative affect and self‐ideal discrepancy after viewing an episode, with the latter increasing further over a season.
- In summary, reality TV model contests have adverse psychological effects on women, which are more pronounced in those with eating disorders, such that these shows may also contribute to the aetiology and maintenance of eating disorders.

## 1 Introduction

Body dissatisfaction is a central psychopathological characteristic of eating disorders (EDs), reflected, for instance, in the desire for a low body weight and restrictive eating behaviours in anorexia and bulimia nervosa (Herpertz‐Dahlmann 2015). Beyond this, body dissatisfaction is a highly prevalent phenomenon in Western cultures. Around half of adult women report dissatisfaction with their bodies (Neumark‐Sztainer et al. 2018), characterised by negative thoughts and feelings about one's body, including size, shape, muscularity, and weight. Body dissatisfaction arises from a perceived discrepancy between the evaluation of one's actual body and the internalised ideal (Grogan 2022), and can be distinguished into trait and state dimensions, with the former being relatively stable over time (Fuller‐Tyszkiewicz et al. 2018). However, numerous studies have shown that body dissatisfaction varies temporally and is influenced by situational factors like exposure to thin beauty ideal images (e.g., Fioravanti et al. 2022). These fluctuations are conceptualised as a state construct (Fuller‐Tyszkiewicz et al. 2018). Furthermore, research has shown that body dissatisfaction is not only a symptom of EDs but also serves as a key predictor of their development (e.g., McLean and Paxton 2019; Stice et al. 2017), with both trait and state dimensions contributing independently (Fuller‐Tyszkiewicz et al. 2018). An empirically well‐supported model to explain the development of body dissatisfaction and further ED symptoms is the tripartite influence model (Roberts et al. 2022; Thompson et al., 1999), which suggests that appearance‐related social comparisons serve as a key mediator in how family, peers, and media affect body dissatisfaction and ED symptoms. Besides appearance pressure through social media, this model includes traditional media, comprising television programs and advertisements, movies, magazines and commercials, as well as music videos (Huang, Peng, and Ahn 2021), with television remaining the medium with the longest daily use in Germany (Harms 2023).

When analysing the portrayal of bodies on television shows, it becomes evident that thin women are overly represented (e.g., Mastro and Figueroa‐Caballero 2018). Media content that emphasises, promotes, or glorifies the thin body type as the ideal standard of beauty is often referred to as thin‐ideal media (e.g., Bessenoff 2006; Harper and Tiggemann 2008). The perception of thin‐ideal media stimuli is thought to involve certain processing mechanisms that are also found in cognitive‐behavioural theories of EDs (Cooper and Fairburn 2010; Fairburn, Cooper, and Shafran 2003; Williamson et al. 2004). These theories provide an approach for understanding how body‐ or food‐related stimuli can influence ED development. Thin‐ideal media stimuli might activate self‐schemas related to body size, shape, or eating, leading to several cognitive biases like attentional bias, an extreme drive for thinness, or overestimation of body size (Williamson et al. 2004). These processes, combined with increased negative affect (e.g., anxiety, depression, or feelings of fatness), can lead to dietary restraint and ED behaviours like restrictive eating, compulsive exercising or body checking, but also binge eating (Williamson et al. 2004). Ultimately, this fosters excessive concerns about body size, internalisation of the thin ideal, fear of fatness, and perfectionism (Williamson et al. 2004). In relation to this model, a recent study illustrated that women's evaluation of body size can be influenced by the frequency with which they see thin bodies (Devine et al. 2022). If this is accompanied by a personal desire to correspond to the depicted body shapes, it can lead not only to body dissatisfaction (Grabe, Ward, and Hyde 2008) but also to negative affect (Aubrey 2007) and low self‐esteem (Bessenoff 2006), which are likewise suggested as risk factors for the development of ED pathology (e.g., Allen et al. 2013; Bakalar et al. 2015). It appears that the body types presented in media might serve as a source of comparison for many women, who evaluate their own body based on the individuals they see on television (Huang, Peng, and Ahn 2021). However, thin‐ideal media consumption appears to have a differential effect on various groups of women, as a meta‐analysis revealed that exposure to thin‐ideal media might exert a negative influence especially on women predisposed to body dissatisfaction (Ferguson 2013).

A prominent representative of thin‐ideal media is the international model contest reality TV franchise *Top Model*, which has aired in over 40 countries worldwide (Schurzmann‐Leder 2021). The aim of each season of the show is to facilitate the entry of a young woman into the fashion industry as a model. The season's winner is rewarded with a non‐cash or cash prize, a picture on the cover of a prominent fashion magazine, and an initially temporary modelling contract with a reputable agency. The ‘next top model’ is determined by a competition lasting for several weeks. The German representative of this format, Germany's Next Topmodel (GNTM), has been an established model contest, with consistently high viewing figures, for almost 20 years (Schurzmann‐Leder 2021). Since the first broadcast in 2006, the show's concept has not significantly changed: Primarily, young female contestants with a thin body shape are cast in the show, and are taught the supposedly fundamental skills of a model ‐ how to use and control their body and how to deploy their personality to optimal effect. Contestants have to prove themselves in model castings and photo shoots, which are usually shown about halfway through each episode. At the end of each episode, the contestants walk on a runway, followed by the jury's decision as to which contestants may proceed to the next stage of the competition. Throughout the episodes, the importance of slimness and beauty is verbally emphasised, and it is also highlighted that appearance and conformity are important factors for recognition and success (Götz 2016). The show is predominantly watched by female teenagers and young adults (Schurzmann‐Leder 2021), an age group for which research indicates the highest incidence rates of anorexia (Javaras et al. 2015) and bulimia nervosa (Stice, Marti, and Rohde 2013).

Regarding the US version of the Top Model format, a qualitative study concluded that an internalisation of the thin body ideal is further promoted by the show's content (McCarthy 2008). Moreover, the treatment of contestants suffering from EDs during the show was criticised (McCarthy 2008). In a study analysing the German version of the show, two thirds of women who were undergoing treatment for an ED reported that GNTM had an at least slight influence on their illness, describing that they compared their bodies with those depicted on the show and realised that their bodies did not correspond to the show's norm (Götz 2016). A further study found that underweight girls who watch GNTM are statistically five times more likely to perceive themselves as overweight compared to individuals who have never watched GNTM (Götz 2018).

Although these studies suggest a possible contribution of such formats to the development and maintenance of body image disturbance and EDs, to date, there is no published empirical study examining the direct effects of reality TV model contest shows like GNTM on female viewers' psychological state. Therefore, the present longitudinal experience sampling study examined the effects of GNTM content on state body dissatisfaction, self‐ideal discrepancy, affect, and self‐esteem in a sample of women with self‐reported EDs as compared to a sample of women without self‐reported EDs. To maximise ecological validity, the participants watched the show in their private environment. Based on the findings of previous research regarding the effects of exposure to thin‐ideal media on women with and without EDs (e.g., Aubrey 2007; Bessenoff 2006; Ferguson 2013; Grabe, Ward, and Hyde 2008; Hausenblas et al. 2013), we expected that participants' diagnostic status would moderate the effects of an episode of GNTM on state body dissatisfaction, self‐ideal discrepancy, negative affect, and self‐esteem, such that a self‐reported ED would be associated with a stronger increase in body dissatisfaction and self‐ideal discrepancy, higher negative affect, and lower self‐esteem (primary hypotheses). Additionally, we hypothesised that diagnostic status would moderate the effect of the whole season of GNTM, such that women with a self‐reported ED would experience a stronger increase in body dissatisfaction and self‐ideal discrepancy compared to those without EDs after watching the entire season (secondary hypotheses).

## 2 Method

### 2.1 Participants

The inclusion criteria for participation in the present study comprised self‐identified female gender and a minimum age of 16 years. The exclusion criteria were current inpatient treatment, acute suicidal ideation, self‐injurious behaviour, current substance abuse, and presence of bipolar, psychotic, or borderline disorder. Additionally, women with EDs could only participate if they would have watched GNTM regardless of the study. In total, *N* = 477 women registered to participate in the study and *n* = 179 women completed the study, of whom *n* = 36 women were suffering from an ED according to self‐report (*n* = 11 women reported anorexia nervosa, *n* = 11 atypical anorexia nervosa, *n* = 3 bulimia nervosa, *n* = 1 atypical bulimia nervosa, *n* = 8 binge‐eating disorder, and *n* = 2 unspecified feeding or eating disorder). Diagnosis was provided by a health professional or psychotherapist in 88.9% of cases, and 58.3% were currently receiving treatment (i.e., outpatient psychotherapy, psychological counselling and/or pharmacotherapy). The mean age was *M* = 28.11 years (range: 20–45 years) in the self‐reported ED group and *M* = 22.06 years (range: 18–52 years) in the group without self‐reported EDs. Participants were recruited via Instagram accounts, e‐mail distribution lists of psychology student councils at German universities, and poster advertisements (e.g., at local universities, an outpatient department, and supermarkets). On Instagram, women with EDs were specifically targeted by posting the study information on specific accounts aimed at this group. Recruitment took place directly before the start of the 18^th^ season of GNTM (January ‐ March 2023), with the study advertised as investigating media effects on body image using the example of GNTM. Both first‐time and regular viewers of GNTM could participate. Regarding highest educational attainment, *n* = 28 women reported having a university degree, *n* = 6 a degree from a university of applied science, *n* = 135 a general qualification for university entrance, *n* = 8 a secondary school certificate, and *n* = 2 selected the response option ‘other ’. Detailed sample characteristics including ED pathology measures are shown in Table 1.

**TABLE 1:** Trait‐like questionnaires and differences between eating disorder group and non‐eating disorder group.

	Eating disorder group			Non‐eating disorder group		
	*N*	*M* (*SD*)	*Min*; *Max*	*N*	*M* (*SD*)	*Min*; *Max*
Body mass index BMI (kg/m^2^)	36	25.47 (11.12)	13.96; 61.81	143	21.77 (3.53)	15.76; 40.22
Age (years)	36	28.11 (7.08)	20; 45	143	22.06 (4.36)	18; 52
EDE‐Q						
Restraint	36	4.87 (1.58)	1.00; 7.00	143	1.86 (1.00)	1.00; 6.20
EDI‐2						
Drive for thinness	36	32.89 (7.21)	17.00; 42.00	143	16.01 (7.15)	6.00; 37.00
Body dissatisfaction	36	44.31 (7.80)	28.00; 54.00	143	24.99 (9.63)	4.00; 49.00

Abbreviations: EDE‐Q = Eating Disorder Examination‐Questionnaire, EDI‐2 = Eating Disorder Inventory‐2, *M* = mean, *Max* = maximum, *Min* = minimum, *N* = sample size, *SD* = standard deviation.

### 2.2 Measures

Participants answered questions referring to demographic characteristics including age, height, weight, educational attainment, mental health diagnoses and their potential treatment, as well as questions concerning ED pathology, body dissatisfaction, affect, and self‐esteem.

#### 2.2.1 Eating Disorder Examination‐Questionnaire

To assess ED‐related psychopathology, we applied the German translation of the Eating Disorder Examination‐Questionnaire (EDE‐Q, Hilbert and Tuschen‐Caffier 2016), focusing on the subscale Restraint, which has shown good internal consistency in the literature (*α* = 0.84, Hilbert, de Zwaan, and Braehler 2012) and in our sample (*α* = 0.83 for women with self‐reported EDs and *α* = 0.76 for women without self‐reported EDs). The convergent validity of the subscale was demonstrated in German‐speaking samples with several different ED diagnoses, as well as non‐clinical and subclinical comparison groups (Hilbert et al. 2007).

#### 2.2.2 Eating Disorder Inventory‐2

Additionally, we used the subscales Body Dissatisfaction and Drive for Thinness from the German version of the Eating Disorder Inventory‐2 (EDI‐2, Paul and Thiel 2004). The internal consistency of both scales can be classified as good according to previous research (0.86 ≤ *α* ≤ 0.89) as well as in our sample (0.84 ≤ *α* ≤ 0.91). The convergent validity of the two subscales was demonstrated in German‐speaking samples that included various ED groups and non‐clinical groups, which were also comparable in age to our sample (Paul and Thiel 2004).

#### 2.2.3 Body Image States Scale

To measure state body dissatisfaction, we utilised the German translation of the Body Image States Scale (BISS, Cash et al. 2002; Vocks, Legenbauer, and Heil 2007). The internal consistency of this questionnaire can be classified as acceptable to excellent according to previous research (0.77 ≤ *α* ≤ 0.90, Cash et al. 2002), and appeared to be higher in our study (*α* = 0.93 for women with self‐reported EDs and *α* = 0.91 for women without self‐reported EDs). The validation of the German translation of the BISS is still pending.

#### 2.2.4 Body Image Matrix of Thinness and Muscularity—Female Bodies

We applied the Body Image Matrix of Thinness and Muscularity—Female Bodies (BIMTM‐FB, Steinfeld et al. 2020) to capture participants' perceived actual body shape as well as their ideal body shape. The self‐ideal discrepancy was calculated as the difference between the perceived and ideal body, with a positive value indicating that the ideal body has a lower body fat percentage than the perceived actual body. The body fat dimension of this figure rating scale has shown good test‐retest reliability (*γ* = 1, Steinfeld et al. 2020). The convergent validity of the body‐fat dimension of the BIMTM‐FB was demonstrated in a German‐speaking sample of comparable age to our sample (Steinfeld et al. 2020).

#### 2.2.5 Positive and Negative Affect Schedule

Additionally, we applied the German version of the Positive and Negative Affect Schedule (PANAS, Krohne et al. 1996; Watson, Clark, and Tellegen 1988) to assess participants' current emotional state, focusing on the Negative Affect subscale, which has shown good internal consistency in the literature (*α* = 0.86, Breyer and Bluemke 2016; Krohne et al. 1996) and in our study (*α* = 0.89 for women with self‐reported EDs and *α* = 0.90 for women without self‐reported EDs). The convergent validity of the PANAS was demonstrated in a representative German sample (Breyer and Bluemke 2016).

#### 2.2.6 Single‐Item Self‐Esteem Scale

Finally, we used the German translation of the Single‐Item Self‐Esteem Scale (G‐SISE, Brailovskaia and Margraf 2020; Robins, Hendin, and Trzesniewski 2001) to measure participants' estimation of their state self‐esteem. As the G‐SISE consists of only one item, no internal consistency can be calculated; therefore, the test‐retest reliability is used as a measure of reliability, which can be classified as good (*γ* = 0.72, Brailovskaia and Margraf 2020). The convergent validity of the G‐SISE was demonstrated in a German‐speaking sample with an age range comparable to that of our sample (Brailovskaia and Margraf 2020).

### 2.3 Materials and Procedure

The online assessment was conducted using the LimeSurvey platform (Version 5.4.14 + 221,205). Participants accessed the survey via a link or QR code. The landing page provided detailed information about the study procedure and outlined the inclusion and exclusion criteria, and participants were asked to give their consent to the processing and storage of personal data and to confirm that none of the mentioned exclusion criteria applied to them. If eligible, participants were asked to complete the first questionnaires, including demographic variables, the EDE‐Q, and EDI‐2. Subsequently, they watched the episodes of the upcoming 18^th^ season of GNTM in their private environment. Participants could choose whether to watch the episodes live, as aired on television weekly, or to stream them at other times. Accordingly, it was also possible for participants to watch several episodes of GNTM in succession. For inclusion of their data in the statistical analyses, participants had to watch at least half of all 18 episodes of the season. To verify this, participants had to specify the date on which each episode they viewed was aired on television. Before the start of each episode, after each episode's photo shoot, and at the end of each episode, participants completed the state questionnaire battery (i.e., BISS, BIMTM‐FB, PANAS, and G‐SISE) on the LimeSurvey platform. To assess participants' adherence, we recorded questionnaire completion times and checked their plausibility regarding timing and intervals between measurements. The first episode of the season was entirely excluded from the survey from the outset, as it differed greatly from the other episodes in terms of content structure. After the end of the season, participants were asked to complete the questionnaires from the first measurement date again. The study was approved by the local ethics committee (vote number 60/2022).

### 2.4 Statistical Analyses

The statistical analyses were conducted with R 4.2 (R Core Team 2022), using the *brms* package (Bürkner 2018) for Bayesian multilevel modelling.

#### 2.4.1 Data Structure

The data are characterised by a multilevel structure with three levels. Level 1 comprises the time of measurement of the four outcome variables (i.e., BISS, BIMTM‐FB, PANAS, and G‐SISE) that participants completed before, during, and after each episode (i.e., pre, during, and post). At level 2, these questionnaires were nested in the episode being watched (i.e., 2, 3, 4, …, 18), and at level 3, the episode was nested within the respective participant (i.e., participant 1, participant 2, …). We tested whether an additional level, namely the day on which the episode was watched, should be included. However, this was not necessary, since the intra‐class correlation for episode number remained small for all outcomes (*ICC* < 0.10).

#### 2.4.2 Missing Values

No values were excluded. On average, participants watched 14.5 episodes (*N =* 2712 observations), which amounts to 85% of all episodes of the season (excluding the first episode). For the episodes that were watched, 95.6% of questionnaires (i.e., pre, during, and post) were completed. Missing values were imputed using random forest imputation via the R package *missForest* (Stekhoven and Bühlmann 2012). The estimated accuracy of the imputed values was very satisfactory for both metric (*NRMSE* < 0.001) and nominal (*PFC* = 0.131) variables.

#### 2.4.3 Data Analytic Strategy

For the primary hypotheses, we conducted three‐level Bayesian multilevel modelling in order to adjust for the hierarchical data structure. This allows for the calculation of robust estimates. The *p*‐values were calculated based on the posterior distribution. The outcome was predicted by the dummy variable time point (levels: pre, during, post; reference level: pre), the binary variable diagnosis (levels: diagnosis, no diagnosis; reference level: no diagnosis), and the interaction thereof. The indices pertain to the time point *i*, the episode *j,* and the participant *k*. The calculation was conducted with a random intercept both for episode ($u_{0jk}$) and for participant ($v_{00k}$). The general formula of the model is as follows:

$$\text{outcom}e_{ijk}=\left(\gamma_{000}+u_{0jk}+v_{00k}\right)+{\gamma_{100}*\text{dia}{\text{gnosis}}_{00k}+\gamma }_{200}*\text{whil}e_{1jk}+\gamma_{300}*\text{whil}e_{1jk}*\text{dia}{\text{gnosis}}_{00k}+\gamma_{400}*\text{pos}t_{2jk}+\gamma_{500}*\text{pos}t_{2jk}*\text{dia}{\text{gnosis}}_{00k}+e_{ijk}$$

This model was calculated for the primary hypotheses by changing the outcome and assessing the significance of the parameter $\gamma_{500}$, which reflects the interaction of time and diagnostic status. For the outcomes state body dissatisfaction and state self‐ideal discrepancy, a Gaussian likelihood function was calculated. For state negative affect, a log‐normal likelihood function was selected based on the variable distribution. Since state self‐esteem is an ordinal variable, ordinal regression was calculated based on a cumulative distribution.

For the secondary hypotheses, a two‐level Bayesian multilevel model was calculated for the levels episodes (level 1) and participants (level 2). The outcome variable was predicted by the episode number, diagnostic status, and the interaction thereof. The outcome was exclusively measured via the questionnaire presented before each episode. Since the *BIC* value for both outcomes (state body dissatisfaction and state self‐ideal discrepancy) was lower for a log‐transformed episode number (state body dissatisfaction: 6877.17 vs. 6878.93; state self‐ideal discrepancy: 5248.9 vs. 5249.0), episode number was log‐transformed. The indices represent the episode *i* and the participant *j*. A random intercept ($u_{0j}$) was calculated for each participant. The model formula is as follows:

$$\text{outcom}e_{ij}=\left(\gamma_{00}+u_{0j}\right)+{\gamma_{10}*\text{dia}{\text{gnosis}}_{0j}+\gamma }_{20}*\text{episode}\_\log_{ij}+\gamma_{30}*\text{dia}{\text{gnosis}}_{0j}*\text{episode}\_\log_{ij}+e_{ij}$$

This model was calculated for both secondary hypotheses by assessing the significance of the parameter $\gamma_{30}$. Again, a Gaussian likelihood function was selected.

Effect sizes were calculated according to Westfall, Kenny, and Judd (2014) by dividing the fixed estimate by the root of the summed variances of the random effects. Effect sizes correspond to Cohen's *d* and can be interpreted accordingly: Effect sizes of 0.20 are considered small, 0.50 moderate, and 0.80 large (Cohen 1988). For the ordinal regression, effect sizes are calculated as odds ratios.

## 3 Results

### 3.1 Primary Hypotheses

Regarding the primary hypotheses, we assumed that after watching an episode of GNTM, women with self‐reported EDs would exhibit a more pronounced increase in state body dissatisfaction, self‐ideal discrepancy, and negative affect, as well as a stronger decrease in self‐esteem compared to women without self‐reported EDs. The hypotheses were confirmed for the outcome variables state body dissatisfaction, self‐ideal discrepancy, and negative affect (*p* < 0.001), with small effect sizes (0.08 ≤ *d* ≤ 0.19). No such effect was found for state self‐esteem. In women without EDs, we only found elevated body dissatisfaction after watching an episode of GNTM (*p* < 0.001). Women with EDs showed significantly higher body dissatisfaction (all *p* < 0.001), self‐ideal discrepancy (all *p* < 0.001), and negative affect (0.0001 ≤ *p* ≤ 0.0031) compared to women without EDs at all measurement time points. Descriptive statistics are reported in Table 2 and test statistics for all four dependent variables are depicted in Table 3. The full models, including all effects, can be found in the Supporting Information S1: Tables S1–S4.

**TABLE 2:** Descriptive values of the outcomes BISS, BIMTM, PANAS‐NA, and G‐SISE.

Primary outcome	Eating disorder group		Non‐eating disorder group		Both	
	*M*	*SD*	*M*	*SD*	*M*	*SD*
BISS pre	6.74	1.56	4.34	1.34	4.85	1.70
BISS during	7.07	1.48	4.52	1.32	5.07	1.71
BISS post	7.18	1.44	4.58	1.33	5.13	1.73
BIMTM pre	2.60	1.30	1.01	1.13	1.35	1.34
BIMTM during	2.74	1.32	0.99	1.11	1.36	1.36
BIMTM post	2.84	1.31	1.00	1.11	1.39	1.38
PANAS‐NA pre	1.90	0.72	1.30	0.49	1.43	0.60
PANAS‐NA during	1.97	0.79	1.31	0.49	1.45	0.63
PANAS‐NA post	2.01	0.81	1.31	0.49	1.46	0.64
G‐SISE pre	2.55	1.42	2.68	1.47	2.66	1.46
G‐SISE during	2.62	1.37	2.67	1.50	2.66	1.47
G‐SISE post	2.52	1.34	2.67	1.49	2.63	1.46

Abbreviations: BIMTM = Body Image Matrix of Thinness and Muscularity, BISS = Body Image States Scale, during = measurement time point during the episode, G‐SISE = German version of the Single‐Item Self‐Esteem Scale, *M* = mean, PANAS‐NA = Positive and Negative Affect Schedule, Negative Affect subscale, post = measurement time point after the episode, pre = measurement time point before the episode, *SD* = standard deviation.

**TABLE 3:** Interaction effect of time and diagnosis for the outcomes BISS, BIMTM, PANAS‐NA, and G‐SISE.

Primary outcome	Estimate	Est. error	*p*‐value (one‐sided)	Effect size *d* (90%‐CI)
BISS	0.201	0.032	< 0.001***	0.14 (0.10; 0.17)
BIMTM	0.241	0.028	< 0.001***	0.19 (0.15; 0.23)
PANAS‐NA	0.038^a^	0.010	< 0.001***	0.08 (0.05; 0.11)
G‐SISE	0.108^b^	0.144	0.227	1.11^c^ (0.88; 1.41)

Abbreviations: 90%‐CI = 90% confidence interval, BIMTM = Body Image Matrix of Thinness and Muscularity, BISS = Body Image States Scale, G‐SISE = German version of the Single‐Item Self‐Esteem Scale, PANAS‐NA = Positive and Negative Affect Schedule, Negative Affect subscale.

^a^ Due to the log‐normal distribution of PANAS‐NA, estimates predict the log‐transformed PANAS‐NA.

^b^ Due to the ordinal level of the G‐SISE, estimates predict log‐odds values.

^c^ Effect size estimates for the G‐SISE are reported as odds ratios.

* indicates *p* ≤ 0.05, ** indicates *p* ≤ 0.01, *** indicates *p* ≤ 0.001.

### 3.2 Secondary Hypotheses

According to our secondary hypotheses, diagnostic status would moderate the effect of the entire season of GNTM on state body dissatisfaction and self‐ideal discrepancy, with an ED diagnosis being associated with stronger effects. Due to the better model fit, the predictor variable ‘episode’ was log‐transformed. No effect was found regarding state body dissatisfaction, meaning that watching one season of GNTM had no lasting influence on state body dissatisfaction. However, for state self‐ideal discrepancy, a significant effect across the whole season was found (*p* < 0.05; see Table 4), with a small effect size (*d* = 0.24), indicating that watching at least half of the season's episodes led female viewers with self‐reported EDs to feel increasingly distant from their body ideal. Test statistics for both dependent variables can be found in Table 4. Full models, including all effects, are depicted in the Supporting Information S1: Table S5.

**TABLE 4:** Interaction effect of episode and diagnosis for the outcomes BISS and BIMTM.

Primary outcome	Estimate	Est. error	*p*‐value (one‐sided)	Effect size *d* (90%‐CI)
BISS	0.009	0.049	0.430	0.02^a^ (−0.15; 0.18)
BIMTM	0.106	0.037	0.002**	0.24^a^ (0.10; 0.38)

Abbreviations: 90%‐CI = 90% confidence interval, BIMTM = Body Image Matrix of Thinness and Muscularity, BISS = Body Image States Scale.

^a^ Effect sizes were calculated across the whole season excluding the first episode, that is 17 episodes.

* indicates *p* ≤ 0.05, ** indicates *p* ≤ 0.01, *** indicates *p* ≤ 0.001.

## 4 Discussion

This longitudinal experience sampling study was the first to empirically examine the impact of the international reality TV show format Top Model, represented by GNTM, on body dissatisfaction and associated constructs across diverse groups. This topic is of notable clinical significance due to the high prevalence of body dissatisfaction in Western societies and the relevance of body dissatisfaction, negative affect, and self‐esteem in the development of EDs (Allen et al. 2013; Bakalar et al. 2015; Neumark‐Sztainer et al. 2018; Roberts et al. 2022; Thompson et al. 1999). Our study design, with high ecological validity, allowed us to examine not only the effects of single episodes of GNTM on state variables but also the impact of an entire season.

The main findings regarding the immediate effect of single episodes indicate that watching GNTM leads to a greater increase in state body dissatisfaction, self‐ideal discrepancy, as well as negative affect in women with self‐reported EDs compared to women without self‐reported EDs. However, both groups showed higher body dissatisfaction after watching GNTM. Moreover, women with self‐reported EDs showed a greater self‐ideal discrepancy and higher negative affect after watching an episode. These negative effects may stem from viewers engaging in constant appearance‐related upward social comparisons with the show's contestants (Götz and Mendel 2017), which may in turn contribute to increased body dissatisfaction and negative affect (Gerber, Wheeler, and Suls 2018; Myers and Crowther 2009). Furthermore, the increase in body dissatisfaction may be related to viewers perceiving their own bodies as larger than those of the contestants, or might be due to a change in viewers' body ideal, which becomes increasingly thinner while watching the show, with both of these factors together potentially leading to a greater state self‐ideal discrepancy. This aligns with the aforementioned study by Devine et al. (2022), in which experimentally increasing the number of presentations of thin bodies led women to more often perceive subsequently shown female bodies as overweight, indicative of the so‐called prevalence‐induced concept change phenomenon. In other words, exposure to episodes of GNTM might result in an inaccurate mental image of what average bodies look like, subsequently leading to their characterisation as unattractive (Devine et al. 2022; Götz 2016). In addition to social comparisons and the prevalence‐induced concept change, the contestants' appearance is subject to commentary and critical assessment by a jury throughout the show. Listening to such messages can increase the internalisation of the thin body ideal and negative affect as compared to listening to body‐positive or neutral talk, even when negative appearance‐related comments refer to other people's bodies (Cruwys, Leverington, and Sheldon 2016; Herbozo and Thompson 2006), potentially further contributing to body dissatisfaction (Liu et al. 2022; Thompson et al. 1999). Our finding of a moderating effect of diagnostic status is in line with previous studies investigating the differential effects of traditional media, especially thin‐ideal media, on women with and without EDs (e.g., Legenbauer, Rühl, and Vocks 2008). It appears that women with EDs, or at least women with a predisposition for EDs, are more reactive and sensitive to this type of media content (Ferguson 2013) and may therefore show a stronger reaction to the Top Model format. This exaggerated response can also be linked to the previously described cognitive‐behavioural model of EDs by Williamson et al. (2004). According to the model, the media stimuli of GNTM might have activated self‐schemas related to body size, shape, or eating in women with EDs, leading to several cognitive biases as well as to increased negative affect. In turn, such processes might not occur to the same extent in women without EDs, which could account for the smaller effects observed in that group.

Contrary to our assumption, participants' self‐esteem was not lower after watching an episode of GNTM than before. However, the current state of research on this topic is inconsistent, with some studies reporting a significant impact of thin‐ideal media on individuals' self‐esteem (e.g., Bessenoff 2006) and others finding no significant effect (e.g., Legenbauer, Rühl, and Vocks 2008). Based on research findings regarding the impact of social media consumption on self‐esteem (Cingel, Carter, and Krause 2022), we assume that self‐esteem is a more stable construct than affect and body dissatisfaction, and therefore subject to fewer fluctuations. Moreover, self‐esteem is influenced not only by one's own body and appearance, but also by factors such as academic performance or positive social relationships (Rentzsch, Wenzler, and Schütz 2016). These factors do not appear to be primarily influenced by the content of GNTM, which may contribute to our non‐significant finding.

Beyond the effects of single episodes, the longitudinal design of the present study enabled us to assess longer‐term changes in participants' experiences over the entire season of GNTM. The analyses revealed that in women with self‐reported EDs, the state self‐ideal discrepancy increased throughout the whole season. As a season of GNTM progresses, women with EDs may feel that they are becoming further away from their body ideal, either due to an increase in perceived body fat of their own body or because the internalised body ideal becomes thinner. It seems that women with EDs do not become desensitised to the show's content over time, but rather that the effects of individual episodes seem to compound over the course of the season, resulting in a sustained impact of GNTM content on the self‐ideal discrepancy. Contrary to our hypothesis, state body dissatisfaction did not show a sustained change over the course of the whole season. This might be due to the different assessment methods, as body dissatisfaction was measured in a text‐based manner whereas the self‐ideal discrepancy was assessed through pictures, which may be more sensitive to change. In addition, the non‐significant finding might be attributable to a ceiling effect, as participants both with and without EDs already indicated high values on the body dissatisfaction scale at the beginning of the season (see Supporting Information S1: Table S5).

Some limitations must be taken into account when interpreting the present findings. First, due to the online format, ED diagnoses were self‐reported, and future studies should therefore validate them using structured interviews. Nevertheless, women with self‐reported EDs scored higher on the EDE‐Q and EDI‐2 compared to those without EDs, indicating that the groups differed in terms of ED symptom severity. Second, the study did not differentiate between different types of EDs. Future research should therefore analyse several ED diagnoses separately, as it is conceivable that the GNTM content may have differential effects on different ED types. Third, we cannot provide any information about the extent to which participants had watched previous seasons of GNTM. It is possible that individuals who had watched GNTM in the past already had stronger body image concerns than those who had never previously watched GNTM and only watched for the purpose of our study. This may have led to a preselection of individuals who participated in the study, meaning that the results should be interpreted with greater caution. Fourth, it would be worthwhile to investigate the effects of thin‐ideal media content on other clinical populations. For instance, as body dysmorphic disorder (BDD) also has an underlying body image component, it seems reasonable to assume that TV shows like GNTM also exert an influence on individuals with BDD. Fifth, the present study lacks data on adolescent girls, who represent a main target group of GNTM. In view of the decreasing age of onset of EDs (Favaro et al. 2019), future studies should ensure that samples of adolescent girls are sufficiently large to enable age to be examined as a further potential variable influencing the results, in addition to the presence of an ED diagnosis. Finally, our findings refer only to the German version of the Top Model format. To evaluate the international generalisability of our results, it would thus be interesting to replicate the present study with the Top Model format from other countries.

Despite these limitations, the present empirical study is the first to investigate the effects of the international Top Model format. By employing an experience sampling design, we were able to capture the effects of GNTM on cognitions and emotions immediately and in participants' private environment, thus increasing the ecological validity of the study and reducing the impact of a possible recall bias (Scollon and Kim‐Prieto 2003). Furthermore, the statistical analysis using Bayesian multilevel regression allowed us to model the hierarchical data structure and the respective outcome distribution, thus yielding more reliable findings than those obtained using other statistical methods. The results of this study highlight the need to sensitise women in general, and groups at high risk of EDs in particular, to the potentially harmful effects of the Top Model content on body dissatisfaction, self‐ideal discrepancy, and negative affect. Women should be informed about these adverse effects to enable them to make informed decisions regarding their consumption of such content. Furthermore, for individuals who have already developed an ED, the influence of thin‐ideal media content on the development and maintenance of symptoms should be evaluated within their treatment, in order to improve media literacy and subsequently reduce any negative effects of thin‐ideal media content (Potter 2013).

## Ethics Statement

The study was approved by the local ethics committee (vote number 60/2022).

## Conflicts of Interest

The authors declare that the research was conducted in the absence of any commercial or financial relationships that could be construed as a potential conflict of interest.

## Supporting information

Supporting Information S1

## Data Availability

The datasets generated for this study are available upon reasonable request to the corresponding first author.
