# Supplementary material for: Assessing the Impact of a Reality TV Fashion Model Contest on Women's State Body Dissatisfaction, Affect, and Self‐Esteem: An Experience Sampling Study of Women With and Without Eating Disorders
Source: Eur Eat Disord Rev. 2025 Mar 5;33(4):815–24. doi: 10.1002/erv.3185 (PMC12171678; doi:10.1002/erv.3185)
Supplement: Supplementary file 1 — Supporting Information S1 [file ERV-33-815-s001.docx]

# Supplementary material

**Table S1**

*Prediction model for BISS*

| Model parameter | Estimate | Est. Error | *p*-value (two-sided) |
| --- | --- | --- | --- |
| Intercept | 4.34 | 0.09 | - |
| Diagnosis | 2.37 | 0.21 | <.001*** |
| During | 0.18 | 0.01 | <.001*** |
| Post | 0.24 | 0.01 | <.001*** |
| During*diagnosis | 0.15 | 0.03 | <.001*** |
| Post*diagnosis | 0.20 | 0.03 | <.001*** |
| $u_{0jk}$^†^ | 0.60 | 0.01 | <.001*** |
| $v_{00k}$^†^ | 1.14 | 0.06 | <.001*** |

*Note.* * indicates *p* ≤ .05, ** indicates *p* ≤ .01, *** indicates *p* ≤ .001, BISS = Body Image States Scale, diagnosis = effect of participant ED diagnosis, during = measurement time point during the episode, post = measurement time point after the episode. ^†^ Random effect estimates are reported as standard deviations. $u_{0jk}$ represents the random intercept for episode and $v_{00k}$ for participant.

**Table S2**

*Prediction model for BIMTM*

| Model parameter | Estimate | Est. Error | *p*-value (two-sided) |
| --- | --- | --- | --- |
| Intercept | 1.00 | 0.08 | - |
| Diagnosis | 1.56 | 0.19 | <.001*** |
| During | -0.02 | 0.01 | 1.00 |
| Post | -0.01 | 0.01 | 1.00 |
| During*diagnosis | 0.15 | 0.03 | <.001*** |
| Post*diagnosis | 0.24 | 0.03 | <.001*** |
| $u_{0jk}$^†^ | 0.39 | 0.01 | <.001*** |
| $v_{00k}$^†^ | 1.00 | 0.05 | <.001*** |

*Note.* * indicates *p* ≤ .05, ** indicates *p* ≤ .01, *** indicates *p* ≤ .001, BIMTM = Body Image Matrix of Thinness and Muscularity, diagnosis = effect of participant ED diagnosis, during = measurement time point during the episode, post = measurement time point after the episode. ^†^ Random effect estimates are reported as standard deviations. $u_{0jk}$ represents the random intercept for episode and $v_{00k}$ for participant.

**Table S3**

*Prediction model for PANAS-NA*

| Model parameter | Estimate | Est. Error | *p*-value (two-sided) |
| --- | --- | --- | --- |
| Intercept | 0.22^‡^ | 0.02 | - |
| Diagnosis | 0.35^‡^ | 0.04 | <.001*** |
| During | 0.00^‡^ | 0.00 | .407 |
| Post | 0.01^‡^ | 0.00 | .163 |
| During*diagnosis | 0.03^‡^ | 0.01 | .006** |
| Post*diagnosis | 0.04^‡^ | 0.01 | <.001*** |
| $u_{0jk}$^†^ | 0.14 | 0.00 | <.001*** |
| $v_{00k}$^†^ | 0.23 | 0.01 | <.001*** |

*Note.* * indicates *p* ≤ .05, ** indicates *p* ≤ .01, *** indicates *p* ≤ .001, PANAS-NA = Positive and Negative Affect Schedule, Negative Affect subscale, diagnosis = effect of participant ED diagnosis, during = measurement time point during the episode, post = measurement time point after the episode. ^†^ Random effect estimates are reported as standard deviations. $u_{0jk}$ represents the random intercept for episode and $v_{00k}$ for participant. ^‡^ Due to the log-normal distribution of PANAS-NA, estimates predict the log-transformed PANAS-NA.

**Table S4**

*Prediction model for G-SISE*

| Model parameter | Estimate | Est. Error | *p*-value (two-sided) |
| --- | --- | --- | --- |
| Intercept[1] | -2.59^‡^ | 0.29 | - |
| Intercept[2] | 0.86^‡^ | 0.28 | - |
| Intercept[3] | 2.09^‡^ | 0.29 | - |
| Intercept[4] | 2.97^‡^ | 0.29 | - |
| Diagnosis | -0.74 | 0.64 | 1.00 |
| During | -0.05 | 0.07 | 1.00 |
| Post | -0.03 | 0.07 | 1.00 |
| During*diagnosis | 0.29 | 0.14 | 0.039* |
| Post*diagnosis | 0.11 | 0.14 | 0.453 |
| $u_{0jk}$^†^ | 2.03 | 0.06 | <.001*** |
| $v_{00k}$^†^ | 3.36 | 0.20 | <.001*** |

*Note.* * indicates *p* ≤ .05, ** indicates *p* ≤ .01, *** indicates *p* ≤ .001, G-SISE = German version of the Single-Item Self-Esteem Scale, diagnosis = effect of participant ED diagnosis, during = measurement time point during the episode, post = measurement time point after the episode. ^†^ Random effect estimates are reported as standard deviations. $u_{0jk}$ represents the random intercept for episode and $v_{00k}$ for participant. ^‡^ Since the G-SISE has five levels, the four intercepts reflect the log odds of a participant falling into, e.g., level ≤ 1 versus level > 1 when the predictor values are set to their reference level (i.e., no diagnosis of eating disorder and pre-episode).

**Table S5**

*Prediction model for BISS and BIMTM across the season*

|  | Model parameter | Estimate | Est. Error | | *p*-value (two-sided) |
| --- | --- | --- | --- | --- | --- |
| BISS | Intercept | 4.28 | | 0.10 | - |
|  | Diagnosis | 2.32 | | 0.23 | <.001*** |
|  | Episode_log | 0.04 | | 0.02 | .098 |
|  | Episode_log*diagnosis | 0.01 | | 0.05 | .860 |
|  | $u_{0j}$^†^ | 1.13 | | 0.06 | <.001*** |
| BIMTM | Intercept | 0.98 | | 0.09 | - |
|  | Diagnosis | 1.36 | | 0.19 | <.001*** |
|  | Episode_log | 0.01 | | 0.02 | .501 |
|  | Episode_log*diagnosis | 0.11 | | 0.04 | .004** |
|  | $u_{0j}$^†^ | 1.00 | | 0.05 | <.001*** |

*Note.* * indicates *p* ≤ .05, ** indicates *p* ≤ .01, *** indicates *p* ≤ .001, BISS = Body Image States Scale, BIMTM = Body Image Matrix of Thinness and Muscularity, diagnosis = effect of participant ED diagnosis, episode_log = log-transformed number of episodes. ^†^ Random effect estimates are reported as standard deviations. $u_{0j}$ represents the random intercept for the participant level.
